# Supplementary material for: Amorphous Blue Phase III: Structure, Materials, and Properties
Source: Materials (Basel). 2024 Mar 11;17(6):1291. doi: 10.3390/ma17061291 (PMC10972530; doi:10.3390/ma17061291)
Supplement: Supplementary file 1 [file materials-17-01291-s001.zip › materials-2893507-supplementary/Video S1/Video S1.pptx]

## Slide 1
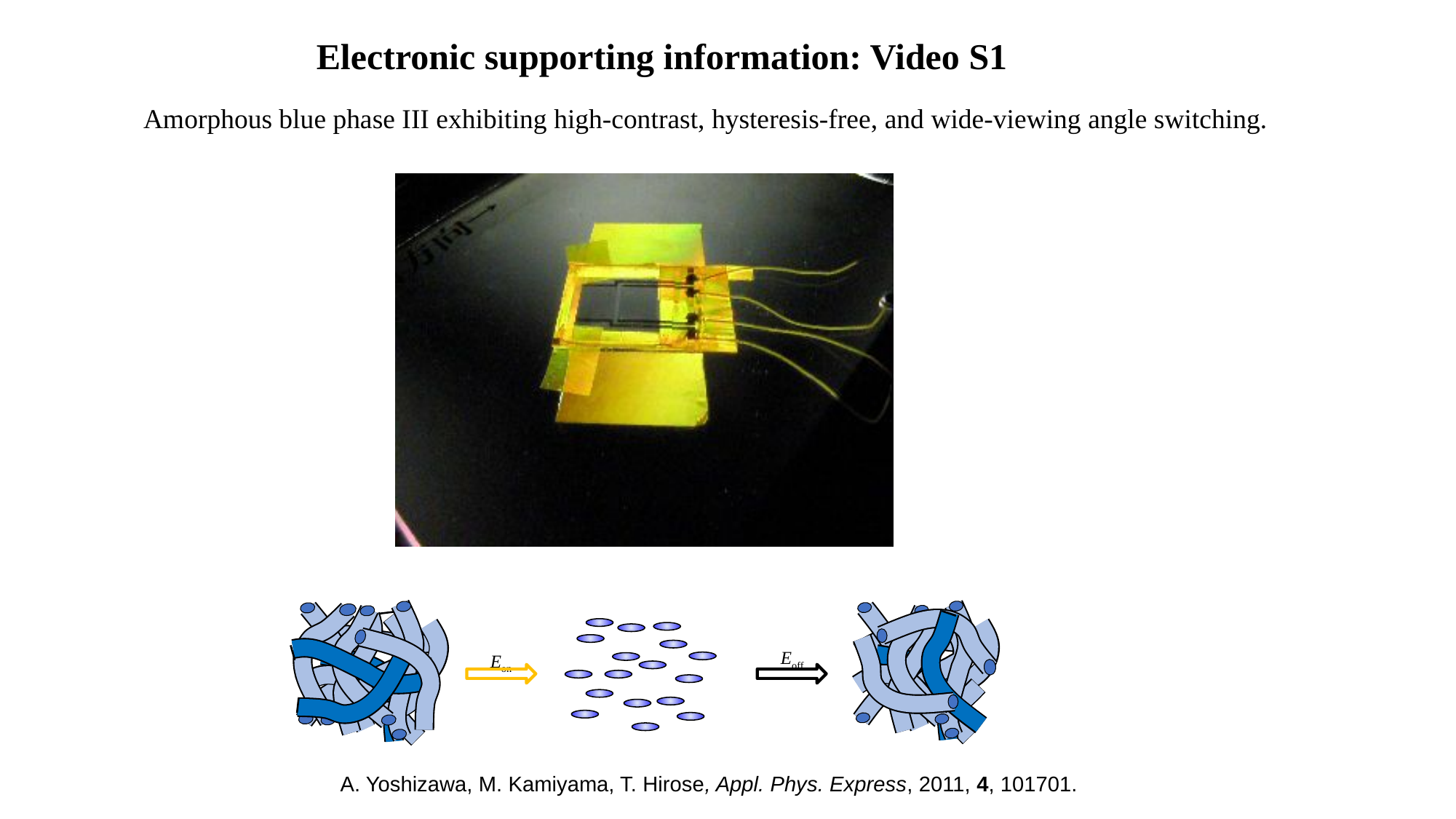

Electronic supporting information: Video S1
Amorphous blue phase III exhibiting high-contrast, hysteresis-free, and wide-viewing angle switching.
Eoff
Eon
A. Yoshizawa, M. Kamiyama, T. Hirose, Appl. Phys. Express, 2011, 4, 101701.
